# Calpain 3 Expression Pattern during Gastrocnemius Muscle Atrophy and Regeneration Following Sciatic Nerve Injury in Rats

**DOI:** 10.3390/ijms161126003

**Published:** 2015-11-11

**Authors:** Ronghua Wu, Yingying Yan, Jian Yao, Yan Liu, Jianmei Zhao, Mei Liu

**Affiliations:** 1 Jiangsu Key Laboratory of Neuroregeneration, Co-Innovation Center of Neuroregeneration, Nantong University, Nantong 226001, China; wuronghua5@ntu.edu.cn (R.W.); 13510003@yjs.ntu.edu.cn (Y.Y.); liuyan@ntu.edu.cn (Y.L.); 2 Department of Histology and Embryology, Medical College, Nantong University, Nantong 226001, China; yjshz@ntu.edu.cn; 3 Department of Pediatrics, the Affiliated Hospital of Nantong University, Nantong 226001, China

**Keywords:** Calpain 3 (CAPN3), gastrocnemius muscle, sciatic nerve injury, myoblast differentiation, rat

## Abstract

Calpain 3 (CAPN3), also known as p94, is a skeletal muscle-specific member of the calpain family that is involved in muscular dystrophy; however, the roles of CAPN3 in muscular atrophy and regeneration are yet to be understood. In the present study, we attempted to explain the effect of CAPN3 in muscle atrophy by evaluating CAPN3 expression in rat gastrocnemius muscle following reversible sciatic nerve injury. After nerve injury, the wet weight ratio and cross sectional area (CSA) of gastrocnemius muscle were decreased gradually from 1–14 days and then recovery from 14–28 days. The active form of CAPN3 (~62 kDa) protein decreased slightly on day 3 and then increased from day 7 to 14 before a decrease from day 14 to 28. The result of linear correlation analysis showed that expression of the active CAPN3 protein level was negatively correlated with muscle wet weight ratio. *CAPN3* knockdown by short interfering RNA (siRNA) injection improved muscle recovery on days 7 and 14 after injury as compared to that observed with control siRNA treatment. Depletion of *CAPN3* gene expression could promote myoblast differentiation in L6 cells. Based on these findings, we conclude that the expression pattern of the active CAPN3 protein is linked to muscle atrophy and regeneration following denervation: its upregulation during early stages may promote satellite cell renewal by inhibiting differentiation, whereas in later stages, CAPN3 expression may be downregulated to stimulate myogenic differentiation and enhance recovery. These results provide a novel mechanistic insight into the role of CAPN3 protein in muscle regeneration after peripheral nerve injury.

## 1. Introduction

Adult skeletal muscle mass and fiber size remain constant under normal conditions. Skeletal muscle atrophy can be induced by a variety of conditions, including starvation, aging, denervation, cancer cachexia, microgravity, and immobilization that can lead to excessive protein degradation [1,2]. Three major protein degradation systems, *i.e.*, ubiquitin–proteasome, autophagy-lysosome, and calpain (CAPN) systems, are thought to contribute to muscle atrophy [3].

CAPN family members are intracellular, calcium-dependent cysteine proteases [4]. Since CAPNs digest many intracellular proteins, their potential to contribute to diseases could be considerable. Unlike the ubiquitously expressed CAPN1 and 2, CAPN3 (also known as p94) is expressed only in skeletal muscle [5,6]. Inactivating mutations in CAPN3 leads to a recessive muscle disorder characterized by progressive weakening and atrophy of proximal limb muscles [6,7,8]. CAPN3 has been reported to hold non-proteolytic functions, such as modulation of calcium efflux in the sarcoplasmic reticulum [9]. CAPN3 function is modulated by phosphorylation of a unique structural insertion [10]. Besides, the unique feature of CAPN3 is the extremely rapid auto-degradation activity [7]. CAPN3 has also been implicated in nuclear factor (NF-κB) signaling in skeletal muscle [11] and plays a role in cancer cell apoptosis [12,13].

Peripheral nerve injury invariably results in the atrophy of the target skeletal muscle, and denervation induces complex changes in the expression of genes involved in muscle fiber atrophy, which is characterized by increased calcium influx. We previously investigated the role of CAPNs in this process by evaluating the messenger RNA (mRNA) expression patterns of *CAPN1*, *2*, and *3* in two models of muscle atrophy induced by sciatic nerve injury or cardiotoxin injection. Our results showed that *CAPN1* and *2* transcript levels were rapidly upregulated and reached a maximum on day seven before decreasing, whereas *CAPN3* mRNA level increased for two weeks and then decreased, in accordance with the process of muscular atrophy and regeneration following injury [14]. Our results of *capn1* and *capn2* expression patterns were in accordance with a previous study [15]. However, *capn3* data was not consistent with the study of Stockholm *et al.* [16] who once reported in 2001 that muscle denervation reduced *CAPN3* mRNA expression in mice. In consideration that mRNA expression may not display similar patterns in different species, or mRNA levels may not be consistent with protein level, we investigated rat CAPN3 gene expression patterns during gastrocnemius muscle atrophy and regeneration following sciatic nerve injury. In the present study, we further applied *CAPN3* gene specific short interfering RNA (siRNA) in rat L6 myoblast cells and injected siRNA into denervated gastrocnemius muscle after sciatic nerve injury to observe the effects of CAPN3.

## 2. Results and Discussion

### 2.1. CAPN3 Is Expressed in the Gastrocnemius Muscle Following Sciatic Nerve Crush Injury

**Figure 1:**
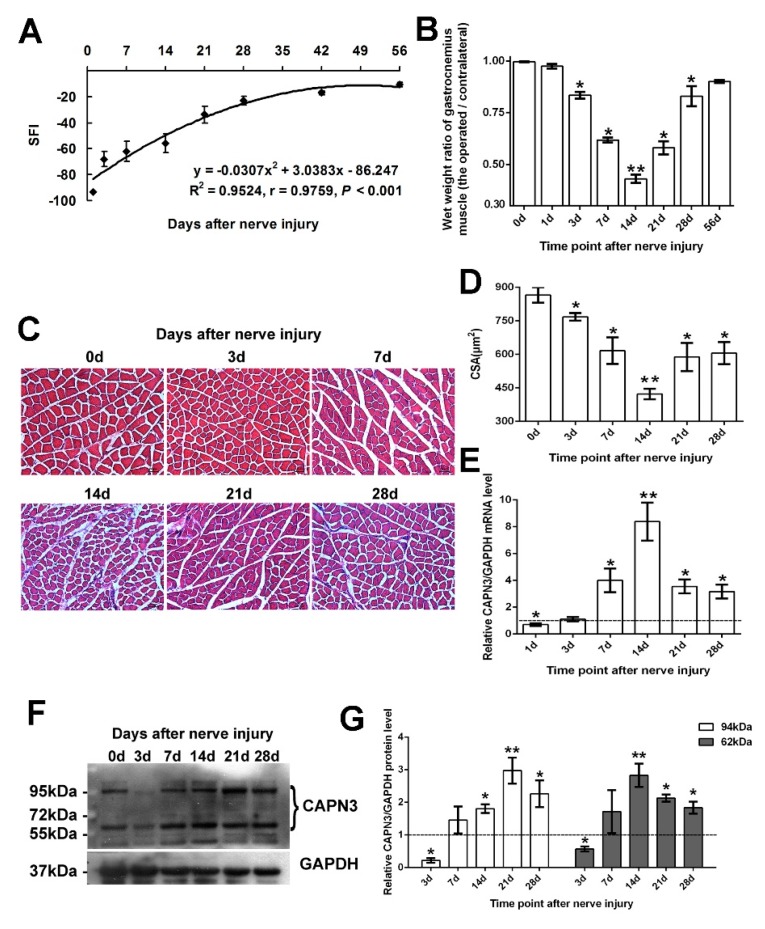
*CAPN3* gene expression in rat gastrocnemius muscle after sciatic nerve crush injury. (**A**–**D**) Reversible sciatic nerve crush injury model. Sciatic function index (SFI) gradually recovered after injury to 86.2% of the control value on day 56 post-injury (**A**) Wet weight ratio (**B**) and Cross sectional area (CSA) of muscle fibers (**C**,**D**) decreased from day 1 to 14 and then increased from day 14 to 28; (**C**) shows the representative pictures visualized by Hematoxylin and Eosin staining (bar = 50 μm); (**D**) shows the statistical results; (**E**) the statistical result of *CAPN3* mRNA expression level by quantitative RT-PCR; (**F**,**G**) shows CAPN3 protein expression; (**F**) result of Western blotting; (**G**) statistical results of latent CAPN3 (~94 kDa) and active CAPN3 (~62 kDa) proteins. Data are expressed as mean ± SEM. * *p* < 0.05, ** *p* < 0.01 *vs.* sham-group (0 day). The data in E, G were normalized, *i.e.*, the average level of relative mRNA or protein of CAPN3 in sham group was set to 1 (which was shown in dotted line), and the average level of CAPN3 in other groups was calculated in relation to this averaged value.

In the reversible sciatic nerve crush injury model, the sciatic function index (SFI) gradually recovered after injury (Figure 1A): on day 56 post-injury, the index had recovered to 86.2% of the control value. After injury, both the wet weight ratio (Figure 1B) and the cross-sectional area (CSA) of muscle fibers (Figure 1C,D) initially decreased (from day 1 to 14) and then increased (from day 14 to 28). Given that the lowest values were observed 14 days post-injury, we assumed day 1 to 14 as the period of muscle atrophy, and day 14 to 28 as the period of regeneration. The mRNA expression of CAPN3 in gastrocnemius muscle was examined on days 1, 3, 7, 14, 21, and 28 after injury. *Capn3* expression declined slightly on day 1 with respect to the control level, but then increased between days 3 and 14 before decreasing up until day 28 (Figure 1E). We examined CAPN3 protein expression using an anti-CAPN3 antibody, which could recognize latent (94 kDa) and active (62 kDa) forms of the protein (Figure 1F,G). Except being reduced on day 3, the level of the 94-kDa CAPN3 protein increased from day 7 to 21, which was followed by a decrease on day 28. Meanwhile, the level of the 62-kDa protein decreased slightly on day 3 and then increased from day 7 to 14 before again decreasing on day 14 to 28.

### 2.2. CAPN3 Expression Is Associated with Gastrocnemius Muscle Atrophy

**Figure 2:**
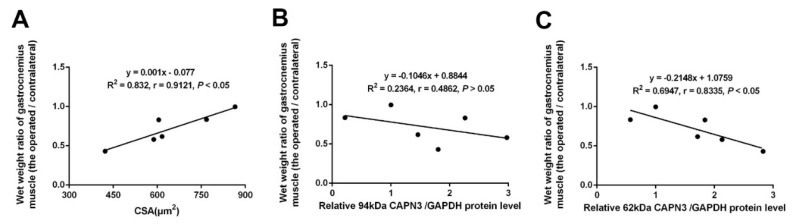
Correlation between CAPN3 expression and the degree of muscle atrophy. (**A**) Linear correlation analysis between wet weight ratio of gastrocnemius muscle and CSA; (**B**,**C**) Linear correlation analysis of wet weight ratio of gastrocnemius muscle with the latent CAPN3 (~94-kDa) protein level (**B**); or the active CAPN3 (~62-kDa) protein level (**C**) from day 0 to 28 after nerve crush injury. *p* < 0.05 was considered significant correlation.

Based on the observed trends in CAPN3 expression, we analyzed the correlation between the dystrophic degree and the level of CAPN3 protein level. We firstly analyzed the linear correlation between wet weight ratio and CSA, and a significant positive correlation was observed (*r* = 0.9121, *p* < 0.05) (Figure 2A). So we chose wet weight ratio as a reference of muscular atrophy. The results showed changes in active CAPN3 (~62-kDa) protein level was significantly and negatively correlated with changes in wet weight ratio (Figure 2C), while there was no correlation observed for the latent CAPN3 (~94-kDa) protein. Expression of the active form of calpain (~62-kDa) was negatively correlated with muscle wet weight, suggesting the activated CAPN3 expression was involved in atrophy of the gastrocnemius muscle following nerve crush injury.

### 2.3. CAPN3 Deficiency Promotes L6 Cell Differentiation and Delays Skeletal Muscle Atrophy

**Figure 3:**
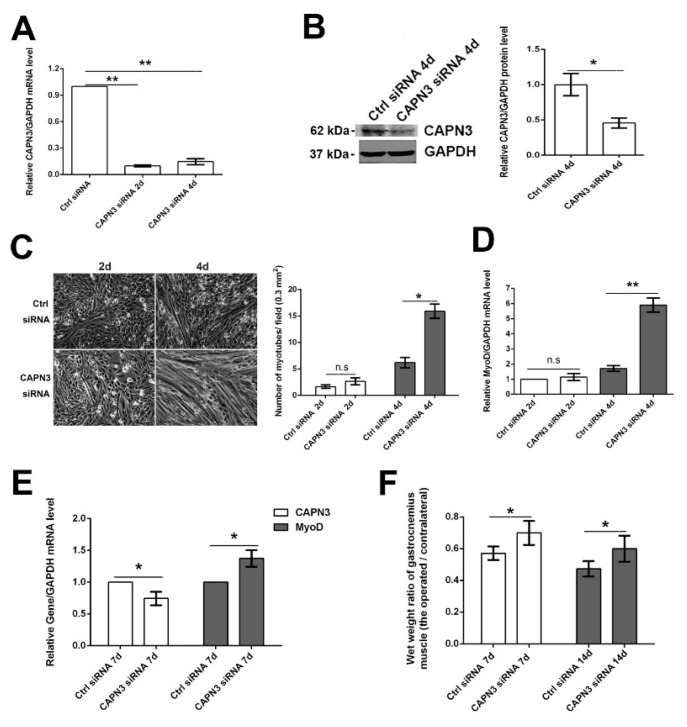
CAPN3 depletion promotes L6 myoblast differentiation and delays gastrocnemius muscle atrophy after sciatic nerve crush injury. (**A**,**B**) Efficiency of CAPN3 siRNA-mediated knockdown was confirmed by quantitative RT-PCR (**A**) and western blotting (**B**); the representative picture of Western blotting and statistical results were shown in **B** (the average CAPN3/GAPDH level of Ctrl siRNA treatment was set to 1); (**C**) L6 myoblast differentiation on days 2 and 4 after CAPN3 siRNA treatment (bar = 50 μm). The representative pictures of differentiation were shown in the left panel of **C**. We took 20 pictures from each slide and calculated number of myotubes in each field. The statistical results of relative myotubes was shown in the right panel of **C**; (**D**) *MyoD* mRNA expression level on day 2 and 4 after CAPN3 siRNA treatment; (**E**) *Capn3* and *MyoD* mRNA level in gastrocnemius muscle on day 7 after CAPN3 siRNA treatment; (**F**) Wet weight ratio of gastrocnemius muscle on day 7 and 14 after CAPN3 siRNA treatment. Data are expressed as mean ± SEM. * *p* < 0.05, ** *p* < 0.01 *vs.* Ctrl siRNA. n.s: Not significant.

To investigate the role of CAPN3 depletion in muscle atrophy/regeneration, we inhibited CAPN3 expression in L6 myoblasts by siRNA knockdown. Compared to Ctrl siRNA-treated cells, CAPN3 siRNA treatment decreased *CAPN3* mRNA levels on days 2 and 4 after transfection (by 90% and 85%, respectively), whereas CAPN3 protein level was decreased by 55% on day 4 (Figure 3A,B). Meanwhile, the number of myotubes increased by 60% and 141% on days 2 and 4, respectively (Figure 3C), and *MyoD* mRNA levels increased by 14% and 440%, respectively (Figure 3D). To examine the function of CAPN3 *in vivo*, siRNA was injected into gastrocnemius muscle following sciatic nerve crush injury. On days 7 and 14 post-injection, the wet weight ratio increased by 23% and 21%, respectively as compared to control rats (*p* *<* 0.05; Figure 3F). We also detected changes in the mRNA levels of *CAPN3* and *MyoD*, *Capn3* mRNA level in CAPN3 siRNA-treated rats decreased by 26%, while *MyoD* transcript level increased by 38% on day 7 (*p* < 0.05; Figure 3E). These results suggest that loss of CAPN3 promotes myoblast differentiation, which may delay gastrocnemius muscular atrophy caused by sciatic nerve crush injury.

Severe peripheral nerve injury often results in target skeletal muscle atrophy [17,18]; however, the mechanisms underlying the degeneration and regeneration of denervated muscle remain unclear. In the present study, we investigated the expression pattern of CAPN3 protein, a member of the calpain family that is particularly expressed in skeletal muscle, during the gastrocnemius muscular atrophy and regeneration. The results showed that at an early stage after sciatic nerve crush, both the mRNA and protein levels of *CAPN3* gene went down compared to the sham group which was likely similar to the result of Stockholm *et al.* [16]. However, after this period, CAPN3 mRNA and protein levels increased gradually during the period of muscle degeneration (day 3–14), and then progressively decreased during the regeneration phase (day 14–28) after reversible sciatic nerve crush injury. The linear regression analysis indicated that the mRNA level and 62-kDa protein expression level of CAPN3 are correlated with the degree of gastrocnemius muscular atrophy. Stuelsatz *et al.* [19] reported that calpain 3 up-regulation negatively affects the differentiation process of C2C12 myoblast cells, and promotes generation of reserve cells in C2C12 myoblasts. Denervation injury reduced the MyoD protein amount [20]. Citing the views of related literature, our results showed an increasing level of CAPN3 expression during muscle degeneration phase, may inhibit myogenic differentiation; in this scenario, the down-regulation of CAPN3 during re-innervation could stimulate myogenic differentiation.

We also detected the effect of *CAPN3* siRNA on rat myoblast L6 cells differentiation and confirmed CAPN3 knockdown increasing the number of fused myotubes. Further, CAPN3 siRNA was injected into the gastrocnemius muscle following denervation. This was supported by the observation that CAPN3 depletion by siRNA-mediated knockdown resulted in a recovery of wet weight ratio by days 7 and 14, which likely reflects increased myoblast differentiation. *MyoD* transcript level was increased at day 7, while *Capn3* mRNA level was decreased.

## 3. Experimental Section

### 3.1. Animals and Surgical Procedures

Sprague Dawley rats, each weighing 200 ± 20 g, were obtained from the Laboratory Animal Center of Nantong University (Nantong, China). Animal surgeries were performed according to institutional animal care and National Institutes of Health (Bethesda, MD, USA) guidelines, and procedures were approved by the Administration Committee of Experimental Animals of Jiangsu Province. Rats were randomly divided into experimental and sham-operated control groups. The former were subjected to sciatic nerve crush injury as described in our previous studies [21,22]. Briefly, 45 rats were anesthetized and the right lateral sciatic nerve was exposed through an incision made mid-thigh on the lateral side of the hind leg and crushed three times using a pair of 12.5-cm microsurgical hemostatic forceps (part no. J31020; Shanghai Medical Instrument Group, Shanghai, China) for 10 s each time at 10-s intervals, while the contralateral nerve was left intact. Then the incisions were closed, and rats were returned to their cages to recover. On predetermined days after surgery, 5 injured rats and 5 sham-operated rats were chosen for SFI detection, and then rats were sacrificed. We removed the gastrocnemius muscles from the operated and contralateral limb. After weighed to determine muscle wet weight, the muscles were frozen in liquid N_2_, and stored at −80 °C. The tissue with a volume of 1.0 × 0.5 × 0.5 cm was obtained from the mid-belly of gastrocnemius muscle and fixed in 4% paraformaldehyde. We treated the control animal in the same manner and designated as the day 0 group.

### 3.2. Functional and Morphometric Analyses Following Sciatic Nerve Crush Injury

As previously described [21,23], the extent of loss or recovery of locomotor function after sciatic nerve crush injury was evaluated by walking-track analysis from each operated group, and was scored using the sciatic function index (SFI), which indicates the degree of nerve dysfunction and varies between 0 and 100. Tests were performed on days 1, 3, 7, 14, 21, 28, 42, and 56 after nerve injury (*n* = 5 rats per group). The SFI for the control group was considered as having a value of zero. For morphometric analyses of gastrocnemius muscle, fixed tissue specimens were washed in water, dehydrated in a graded ethanol series, cleared in xylene, embedded in paraffin, and cut into 5-μm-thick sections. Hematoxylin and Eosin (HE) staining was carried out on every sixth section and was visualized by light microscopy. Images from six random fields were acquired for the sixth stained sections of each specimen using a DC 300F color digital camera (Q550 IW; Leica Imaging Systems, Cambridge, UK) and then digitized with the affiliated image analysis system; the cross-sectional area (CSA) of muscle fibers was measured with QWin software (Leica).

### 3.3. CAPN3 Short Interfering (si) RNA Knockdown

The siRNAs used in this study were purchased from Biomics Biotechnologies Inc. (Nantong, China). Sequences of the universal control (Ctrl) and gene-specific *CAPN3* siRNAs are listed in Table 1. The siRNAs were modified with a 5′ CH_3_O-group that provided stability for long-term *in vivo* studies. The siRNAs were dissolved in RNase-free H_2_O at a concentration of 0.33 μg/μL and mixed with Lipofectamine 2000 (Invitrogen, Shanghai, China) at a ratio of 4 μL Lipofectamine to 1 μg siRNA in a total volume of 50 μL. The Lipofectamine-siRNA complex was injected into denervated gastrocnemius muscle using a Quintessential Stereotaxic Injector (Stoelting, Wood Dale, IL, USA). On day 3 after the surgery, injured rats were randomly divided into two groups (CAPN3 siRNA group and Ctrl siRNA group), anesthetized, and the right lateral gastrocnemius muscle was exposed. The Lipo-siRNA mixture was injected into five sites (10 μL per site over a period of 2 min, with the needle left in place for an additional minute to prevent spillover) at a depth of 2.0 mm. Rats were injected with Ctrl or CAPN3 siRNA (*n* = 10 rats per group). The injection was immediately followed by the delivery of eight 20-ms pulses at 200 V/cm and 1 Hz using caliper electrodes placed across the injected leg and connected to an ECM-830 electroporation device (BTX, Holliston, MA, USA), as previously described [24,25]. In L6 cells, siRNAs were transfected using Lipofectamine 2000 following the manufacturer’s instructions. For analysis the effect of siRNA treatment on myoblast differentiation, the transfection mediun was changed by differentiation medium after siRNA transfection for 6 h.

**Table 1:** Oligonucleotides used in this study.

Usage	Target	Sequence (5′ to 3′)
qRT-PCR	*GAPDH* sense	CCTTCATTGACCTCAACTACATG
	*GAPDH* antisense	TCAAACTTGTGATCCAGGCG
	*CAPN3* sense	CATTGTCCCCTCCACTTACG
	*CAPN3* antisense	GCTCCTTGTTGCTGTTTGC
	*MyoD* sense	CGACTCTTCAGGCTTGGGTT
	*MyoD* antisense	CCAGGTCCTCAAAAAAGCG
siRNA sequence	*Ctrl* sense	CCUACGCCACCAAUUUCGUTT
	*Ctrl* antisense	ACGAAAUUGGUGGCGUAGGTT
	*CAPN3* sense	CUGAAGACAAGGGUUCATT
	*CAPN3* antisense	UGAACCCUUGUGUCUUCAGTT

### 3.4. Real-Time (RT-) PCR and Western Blot Analysis

Total RNA was extracted with TRIzol reagent (Invitrogen) and used to synthesize cDNA with an Omniscript RT kit (Qiagen, Hilden, Germany) following the manufacturer’s instructions. Sequences of primers used to amplify *CAPN3*, *MyoD*, and glyceraldehyde-3-phosphate dehydrogenase (*GAPDH*; used as an internal control) are shown in Table 1. Western blotting was performed according to our standard protocols [26] using the following antibodies: goat anti-GAPDH polyclonal (1:800; Santa Cruz Biotechnology, Santa Cruz, CA, USA), rabbit anti-CAPN3 polyclonal (1:400; Millipore Cat. No. AB81019, detecting latent and activated forms of CAPN3, Billerica, MA, USA), donkey anti-goat IRDye (1:10,000; Rockland, Limerick, PA, USA), and donkey anti-rabbit IRDye (1:10,000; Rockland). Immunoblots were analyzed using the Odyssey densitometry program (LI-COR, Lincoln, NE, USA). GAPDH was used as a loading control.

### 3.5. L6 Cell Culture, Differentiation and Myotube Calculation

L6 rat myoblast cells (ATCC) were cultured in the recommended medium (Dulbecco’s Modified Eagle’s Medium with 10% fetal bovine serum) under standard conditions. To induce differentiation, cells were cultured in differentiation medium consisting of Dulbecco’s Modified Eagle’s Medium with 2% horse serum instead of 10% fetal bovine serum; differentiation was examined daily, followed by visualization by microscopy (Leica, Wetzlar, Germany). The round cells with single nucleus changed into myotube cells with multiple nuclei gradually. The mean number of myotubes was determined from 10 random fields.

### 3.6. Statistical Analysis

All experiments were performed at least three times. Data are expressed as mean ± SEM and were analyzed by one-way analysis of variance and the unpaired Student’s *t* test when necessary. *p* < 0.05 was considered statistically significant.

## 4. Conclusions

In summary, the results of this study demonstrate that the CAPN3 expression pattern correlates with the degree of muscle atrophy and regeneration after sciatic nerve injury in rats. High levels of CAPN3 are expressed in muscle atrophic phase after denervation, while subsequent downregulation of CAPN3 expression is found in the muscular regeneration phase. Depletion of *CAPN3* gene expression could promote myoblast differentiation and delay muscle atrophy. Based on these results, we suggest that CAPN3 expression is upregulated during muscle atrophy, which may promote satellite cell renewal by inhibiting myogenic differentiation; however, in later stages, CAPN3 expression is downregulated, which stimulates myogenic differentiation and enhances recovery. Our study supports the role of CAPN3 during skeletal muscle atrophy and regeneration by reason of peripheral nerve injury. These findings may also provide a mechanistic insight into muscle regeneration that can potentially be applied to the development of therapeutics to treat certain types of muscular dystrophy.
